# Protein Never in Mitosis Gene A Interacting-1 regulates calpain activity and the degradation of cyclooxygenase-2 in endothelial cells

**DOI:** 10.1186/1476-9255-6-20

**Published:** 2009-06-22

**Authors:** Tongzheng Liu, Ryan A Schneider, Vaibhav Shah, Yongcheng Huang, Rostislav I Likhotvorik, Lakhu Keshvara, Dale G Hoyt

**Affiliations:** 1 Division of Pharmacology, The Ohio State University College of Pharmacy, and The Dorothy M. Davis Heart and Lung Research Institute, Columbus, Ohio 43210, USA.; 2 Department of Molecular Genetics, University of Texas Southwestern Medical Center at Dallas, 5323 Harry Hines Blvd, Dallas, Texas 75390, USA.

## Abstract

**Background:**

The peptidyl-proline isomerase, Protein Never in Mitosis Gene A Interacting-1 (PIN1), regulates turnover of inducible nitric oxide synthase (iNOS) in murine aortic endothelial cells (MAEC) stimulated with *E. coli *endotoxin (LPS) and interferon-γ (IFN). Degradation of iNOS was reduced by a calpain inhibitor, suggesting that PIN1 may affect induction of other calpain-sensitive inflammatory proteins, such as cyclooxygenase (COX)-2, in MAEC.

**Methods:**

MAEC, transduced with lentivirus encoding an inactive control short hairpin (sh) RNA or one targeting PIN1 that reduced PIN1 by 85%, were used. Cells were treated with LPS/IFN, calpain inhibitors (carbobenzoxy-valinyl-phenylalaninal (zVF), PD150606), cycloheximide and COX inhibitors to determine the effect of PIN1 depletion on COX-2 and calpain.

**Results:**

LPS or IFN alone did not induce COX-2. However, treatment with 10 μg LPS plus 20 ng IFN per ml induced COX-2 protein 10-fold in Control shRNA MAEC. Induction was significantly greater (47-fold) in PIN1 shRNA cells. COX-2-dependent prostaglandin E2 production increased 3-fold in KD MAEC, but did not increase in Control cells. The additional increase in COX-2 protein due to PIN1 depletion was post-transcriptional, as induction of COX-2 mRNA by LPS/IFN was the same in cells containing or lacking PIN1. Instead, the loss of COX-2 protein, after treatment with cycloheximide to block protein synthesis, was reduced in cells lacking PIN1 in comparison with Control cells, indicating that degradation of the enzyme was reduced. zVF and PD150606 each enhanced the induction of COX-2 by LPS/IFN. zVF also slowed the loss of COX-2 after treatment with cycloheximide, and COX-2 was degraded by exogenous μ-calpain *in vitro*. In contrast to iNOS, physical interaction between COX-2 and PIN1 was not detected, suggesting that effects of PIN1 on calpain, rather than COX-2 itself, affect COX-2 degradation. While cathepsin activity was unaltered, depletion of PIN1 reduced calpain activity by 55% in comparison with Control shRNA cells.

**Conclusion:**

PIN1 reduced calpain activity and slowed the degradation of COX-2 in MAEC, an effect recapitulated by an inhibitor of calpain. Given the sensitivity of COX-2 and iNOS to calpain, PIN1 may normally limit induction of these and other calpain substrates by maintaining calpain activity in endothelial cells.

## Background

Protein Never in Mitosis Gene A Interacting-1 (PIN1) is an enzyme that regulates transcription, and turnover of mRNA and proteins. PIN1 is a *cis-trans *peptidyl-prolyl isomerase that contains an amino-terminal domain, the tryptophan-tryptophan (WW) domain, which is characterized by two tryptophan residues separated by 22 amino acids that can bind to phosphorylated serine- or threonine-proline sequences in substrate proteins. PIN1 also isomerizes this motif with its carboxy-terminal catalytic domain [1]. Isomerization of the phosphorylated serine- or threonine-proline motif has a significant effect on conformation of many phospho-proteins. The conformational switching catalyzed by PIN1 allows it to regulate transcription factors, mRNA stabilization factors, and the susceptibility of a growing list of proteins to post-translational modifications and proteases [1-5].

Previously, we found that depletion of PIN1 and treatment with a calpain inhibitor each reduced the degradation of inducible nitric oxide synthase (iNOS) in murine aortic endothelial cells (MAEC) stimulated with *E. coli *endotoxin (LPS) and interferon-γ (IFN). PIN1 bound to iNOS suggesting that it might directly regulate the sensitivity of iNOS to calpain [6]. PIN1 may also regulate expression of inflammatory proteins by an effect on calpain.

Cyclooxygenase (COX)-2 is induced by LPS, IFN, and other factors in endothelial cells cultured from various organs and species [7-14]. Elevated endothelial COX-2 may contribute to vascular pathogenesis [15,16]. This enzyme is also significant for endotoxin action as COX-2 knockout mice are resistant to LPS-induced inflammation and death [17]. COX-2 has a relatively short half-life, indicating that turnover may effectively control its expression [8]. While COX-2 and iNOS can be degraded by several processes [6,8,18-20], calpain inhibitors are known to suppress cleavage of iNOS [6] and COX-2 [18].

The purpose of this investigation was to determine whether PIN1 regulates the expression of COX-2, which is induced by LPS and IFN in MAEC. It was hypothesized that PIN1 would associate with COX-2 and that depletion of PIN1 would enhance its induction in MAEC. The impact of PIN1 depletion on calpain activity was also determined.

## Methods

Endothelial cell growth supplement, heparin, phenylmethylsulfonyl fluoride, Bradford reagent, *E. coli *LPS, serotype 0111:B4, and arachidonic acid were obtained from Sigma Chemical Co. (St Louis, MO). Recombinant mouse IFN was from R&D Systems (Minneapolis, MN). Cycloheximide, carbobenzoxy-valinyl-phenylalaninal (zVF, MDL-28170 or calpain inhibitor III), PD150606, porcine μ-calpain, [4-((4-(dimethylamino)phenyl)azo)benzoic acid, succinimidyl ester]-threonine-proline-leucine-lysine~serine-proline-proline-proline-serine-proline-arginine-[5-((2-aminoethyl)amino)naphthalene-1-sulfonic acid], and carboxybenzyl-phenylalanine-arginine-7-amido-4-methylcoumarin were obtained from Calbiochem (La Jolla, CA). Fetal bovine serum was from Hyclone Laboratories (Logan, UT). Agarose, ethidium bromide, ethylenediamine tetraacetic acid, sodium dodecyl sulfate, NaCl, Na_3_VO_4_, NaF, tris-base and tween 20 were obtained from Fisher Scientific (Fair Lawn, NJ). Triton X-100 was from Pierce (Rockford, IL). Dulbecco's minimum essential medium, trypsin, Trizol, Superscript Reverse Transcriptase Taq DNA polymerase, RNAse-free DNAse, deoxynucleotides, and protein G agarose were purchased from Invitrogen (Carlsbad, CA). Glutathione-sepharose was purchased from Amersham Biosciences (Uppsala, Sweden). A prostaglandin E2 competition enzyme-linked immunosorbent assay kit was obtained from R and D Systems, Minneapolis, MN. Anti-COX-2 antibody directed against a 16 amino acid sequence, ending 7 residues from the C-terminus of the protein, SC-560 and NS-398 were purchased from Cayman Chemical (Ann Arbor, MI). Anti-PIN1 was from R&D Systems (Minneapolis, MN). Horseradish peroxidase-conjugated goat anti-mouse and goat anti-rabbit secondary antibodies were from Jackson Immunoresearch Laboratories, Inc. (West Grove, PA). Renaissance Enhanced Chemiluminescence Reagent was purchased from New England Nuclear Life Sciences (Boston, MA).

### Cells

MAEC were cultured from aortas of mice in accordance with the Guide for the Care and Use of Laboratory Animals from the U.S. National Institutes of Health [21]. As described previously, cells were transduced with short hairpin RNA (shRNA) to knockdown (KD) PIN1 or with an inactive mutant sequence (Control), and selected for stable modification. This produced KD MAEC with approximately 15% of the level of PIN1 protein found in Control and non-transduced MAEC [6].

### Treatments

KD and Control MAEC were incubated in Dulbecco's minimum essential medium/0.5% fetal bovine serum for 18 h, and then treated with medium or 10 μg LPS and 20 ng IFN per ml, and other agents for various times. zVF or PD150606 were added 1 h before LPS/IFN to inhibit calpain [22]. Ninety μg cycloheximide/ml was used to inhibit protein synthesis after induction of COX-2 with LPS/IFN [6]. COX-2-dependent prostaglandin E2 production was measured after incubating cells with LPS/IFN for 24 h. Cells were then incubated in fresh medium containing 20 μM arachidonic acid, LPS, IFN and the COX-1 selective antagonist, SC-560 (1 μM) [23], with or without the COX-2 selective antagonist, NS-398 (10 μM) [24]. The medium was collected after 2 h and stored at -80 degrees C. Prostaglandin E2 was measured by competition enzyme-linked immunosorbent assay in comparison with prostaglandin E2 standard by the manufacturer's instructions.

### mRNA Levels

RNA was extracted with Trizol, precipitated, and dissolved in water. cDNA was produced from 3 μg of RNA. cDNA was amplified by polymerase chain reaction for β-actin as described previously [25], and for COX-2. COX-2 primers were sense, 5'-CCG GAC TGG ATT CTA TGG TG, and antisense, 5'-AGG AGA GGT TGG AGA AGG CT from Genbank accession BC052900, producing a 263 base pair product. Half of each reaction was electrophoresed in 1% agarose. Gels were imaged and analyzed after ethidium bromide staining [25].

### Immunoprecipitation, glutathione s-transferase pulldown, and Western blotting

As previously described [6], cells were washed, sonicated in lysis buffer, and protein concentration was measured. For western blotting, 12 μg of sample protein were denatured and separated on 4–20% Tris-gylcine, SDS-polyacrylamide gels and transferred to nitrocellulose. For immunoprecipitation, 500 μg of cell lysate protein was incubated with 5 μg anti-PIN1 antibody and protein G agarose. For pulldown, glutathione S-transferase or glutathione S-transferase-PIN1 fusion protein was added to 500 μg of cell lysate protein and glutathione-sepharose. Samples were then denatured for electrophoresis and western blotting. Blots were immunostained and imaged on X-ray film by enhanced chemiluminescence. Films were scanned and digital images of proteins were analyzed.

### Calpain activity

Calpain activity was measured as described by Tompa et al. [26]. Cells were washed with and scraped in 1 ml of ice-cold PBS, and collected by centrifugation (1500 × g, 2 min at 4°C). Collected cells were resuspended in 100 mM Tris-HCl, 5 mM EDTA, 1 mM dithiothreitol, 5 mM benzamidine, 0.5 mM phenylmethylsulfonyl fluoride, and 10 mM β-mercaptoethanol, and sonicated four times for 10 s at 4°C with 1 min pauses in between. The lysate was centrifuged at 15,000 × g for 20 min at 4°C to remove the cell debris. Calpain activity was measured with the fluorogenic calpain substrate, [4-((4-(dimethylamino)phenyl)azo)benzoic acid, succinimidyl ester]-threonine-proline-leucine-lysine~serine-proline-proline-proline-serine-proline-arginine-[5-((2-aminoethyl)amino)naphthalene-1-sulfonic acid]. The reaction mixture contained 40 μg protein and 15 mM calcium in 50 μl of buffer (10 mM HEPES, 150 mM NaCl, 1 mM EDTA, 5 mM benzamidine, 0.5 mM phenylmethylsulfonyl fluoride, 10 mM β-mercaptoethanol, pH 7.5). The reaction at 30°C was started by adding substrate to 100 μM. The initial velocity of increasing fluorescence, using 320 nm excitation and 480 nm emission, was determined.

The susceptibility of COX-2 to degradation by exogenous calpain *in vitro *was also determined. Extracts were incubated in calpain reaction buffer as above in the presence of porcine μ-calpain for 30 min at 30°C. Reactions were stopped by addition of denaturing sample buffer and subjected to western blotting as described above.

### Cathepsin activity

Cells were washed three times with PBS and scraped in 1 ml of ice-cold PBS. Cells were collected by centrifugation at 1500 × g for 2 min at 4°C. The pellet was resuspended in reaction buffer (50 mM Na-acetate, 1 mM EDTA and 2 mM dithioerythritol pH 5.5), and sonicated four times for 10 s with 1 min breaks. The lysate was centrifuged at 1500 × g for 5 min at 4°C to remove debris. The reaction was started by mixing 20 μM cathepsin substrate, carboxybenzyl-phenylalanine-arginine-7-amido-4-methylcoumarin, in the reaction containing 2 μg supernatant protein, as described by Werle et al. [27]. 7-amido-4-methylcoumarin release was monitored at 37°C for 30 min by fluorescence, with excitation at 380 nm and emission at 460 nm, and the initial velocity was determined.

### Data analysis

Bands in images of polymerase chain reaction gels and scanned western blots were measured with Image J 1.34 s (NIH). Prostaglandin E2 concentrations were estimated from a standard curve and calpain activity was indicated by the fluorescence increase per minute. Data were analyzed by Student's *t *test or analysis of variance with Bonferroni correction for multiple comparisons [28].

## Results

**Figure 1:**
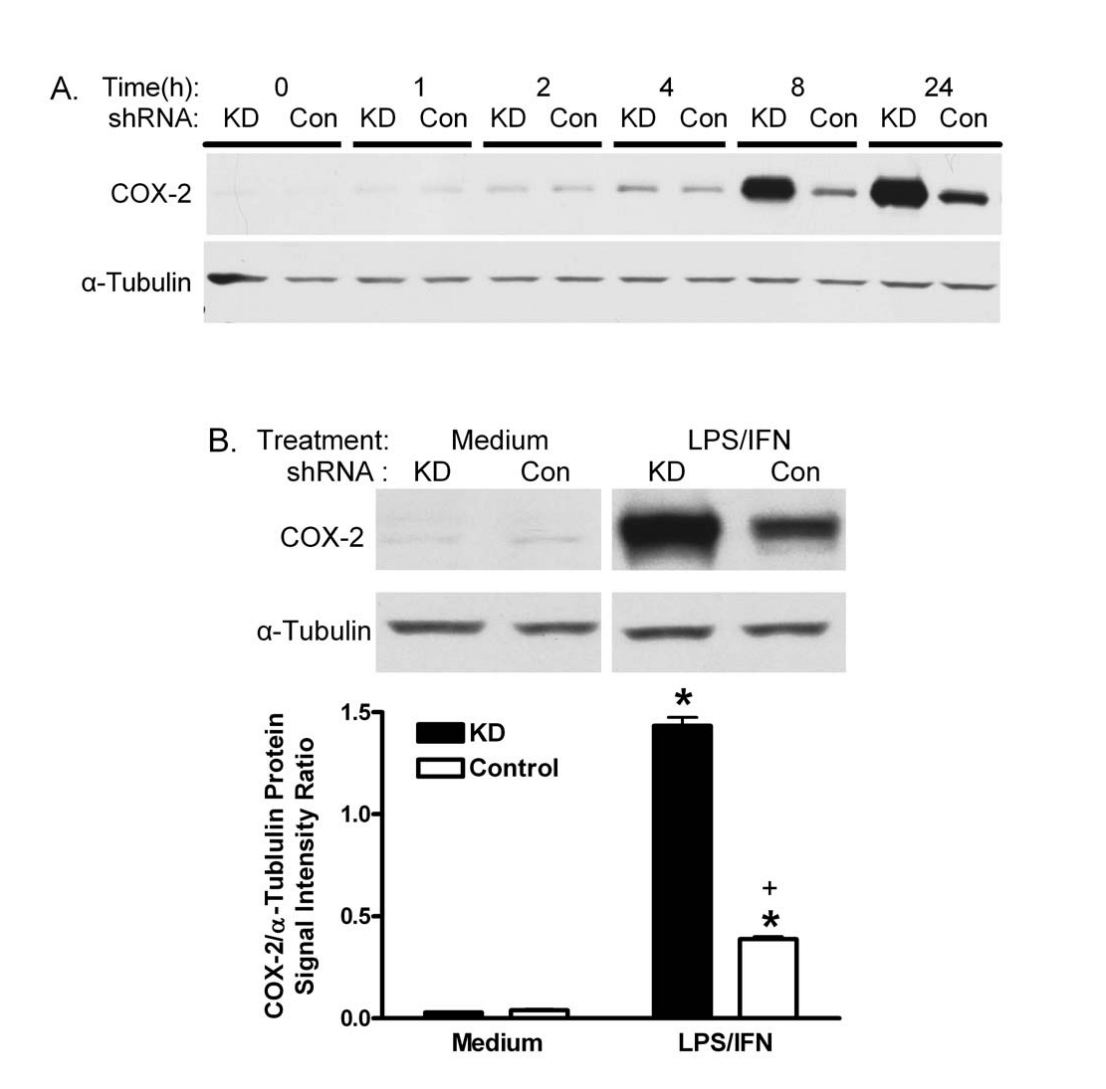
**Effect of PIN1 knockdown on COX-2 protein**. A, KD and Control (Con) shRNA MAEC were treated with LPS and IFN for 0–24 h. B, Cells were treated with medium or LPS and IFN for 24 h. Representative western blots of COX-2 and α-tubulin are shown. Bars in B represent mean + SE ratio of COX-2/α-tubulin from densitometric analysis of 3 cultures of each group. *:p < 0.05 for comparison between medium- and LPS/IFN-treated cells, and +, p < 0.05 for comparison between KD and Control cells treated in the same manner.

**Figure 2:**
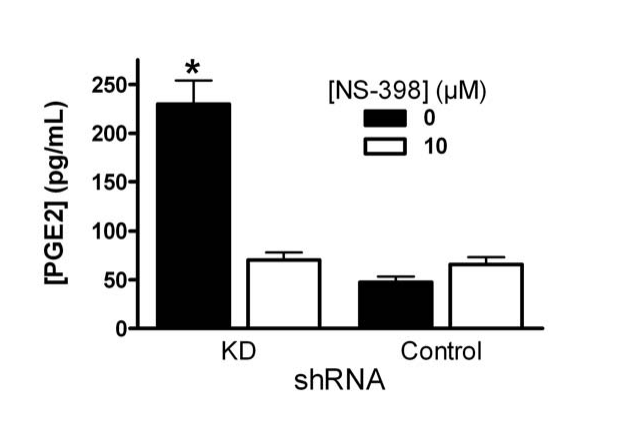
**Effect of PIN1 knockdown on prostaglandin E2**. COX-2-dependent prostaglandin E2 (PGE2) production was measured in cells treated with LPS, IFN, 20 μM arachidonic acid and 1 μM SC-560, with or without 10 μM NS-398. Bars represent mean + SE concentration of prostaglandin E2 in medium for 5 cultures in each group. *:p < 0.05 for between KD and Control cells treated in the same way.

**Figure 3:**
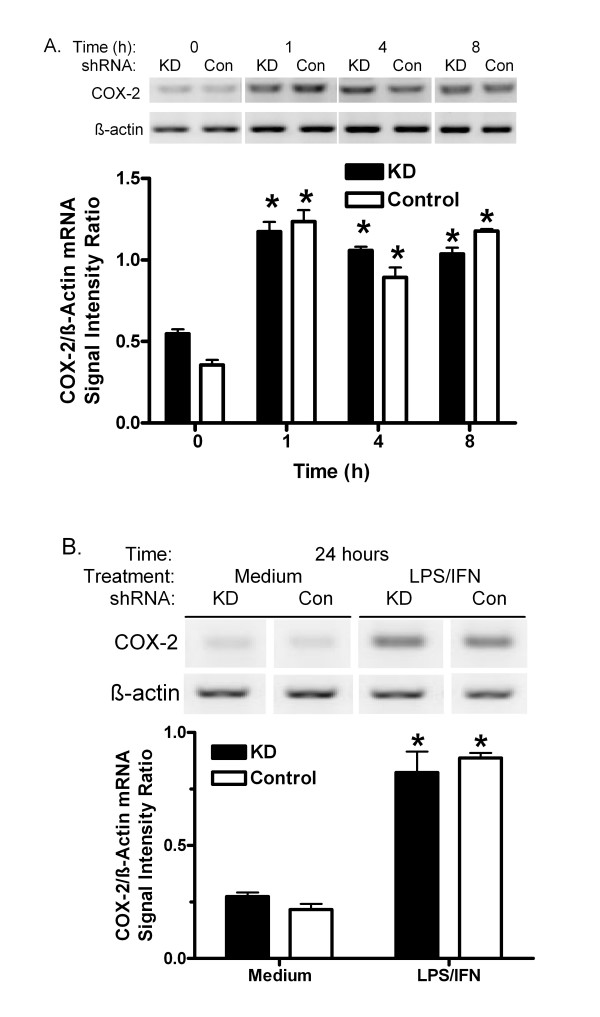
**Effect of PIN1 knockdown on COX-2 mRNA**. A, KD and Control (Con) shRNA MAEC were treated with LPS and IFN for 0–8 h. B, Cells were treated with medium or LPS and IFN for 24 h. mRNAs encoding COX-2 and β-actin were determined by RT-PCR and agarose gel electrophoresis. Representative ethidium bromide-stained gels are shown. Replicate COX-2 or β-actin products in a single gel were imaged for analysis. Bars represent mean + S.E. ratio of COX-2: β-actin products from densitometric analysis of images from 3 independent cultures. *:p < 0.05 for comparison with cells treated for 0 h in A, or with medium in B.

Previously, KD shRNA was shown to reduce PIN1 by 85% compared with Control shRNA in MAEC [6]. COX-2 protein was very low in vehicle-treated KD and Control MAEC (figure 1), and incubation with either LPS or IFN alone did not induce it (data not shown). However, stimulation with 10 μg LPS plus 20 ng IFN per ml increased COX-2 expression. The protein appeared to increase as early as 1 h after treatment, and induction persisted through 24 h. Differences between KD and Control cells were qualitatively noticeable by 4 h after treatment and became greater with time (figure 1A). After 24 h, the signal for COX-2 protein was increased 10-fold in Control shRNA MAEC (figure 1B). The COX-2 signal was significantly more induced in PIN1 KD cells (47-fold). Similar results were obtained in 2 other independent pairs of cultures selected for the KD and Control shRNA (data not shown). COX-2-mediated prostaglandin E2 production increased 3-fold in KD MAEC, but not in the Control cells (figure 2). LPS/IFN induced COX-2 mRNA in KD and Control shRNA cells as indicated by RT-PCR. The message increased within 1 h and remained elevated at 24 h. However, there was no difference between KD and Control shRNA MAEC at any time (figure 3).

**Figure 4:**
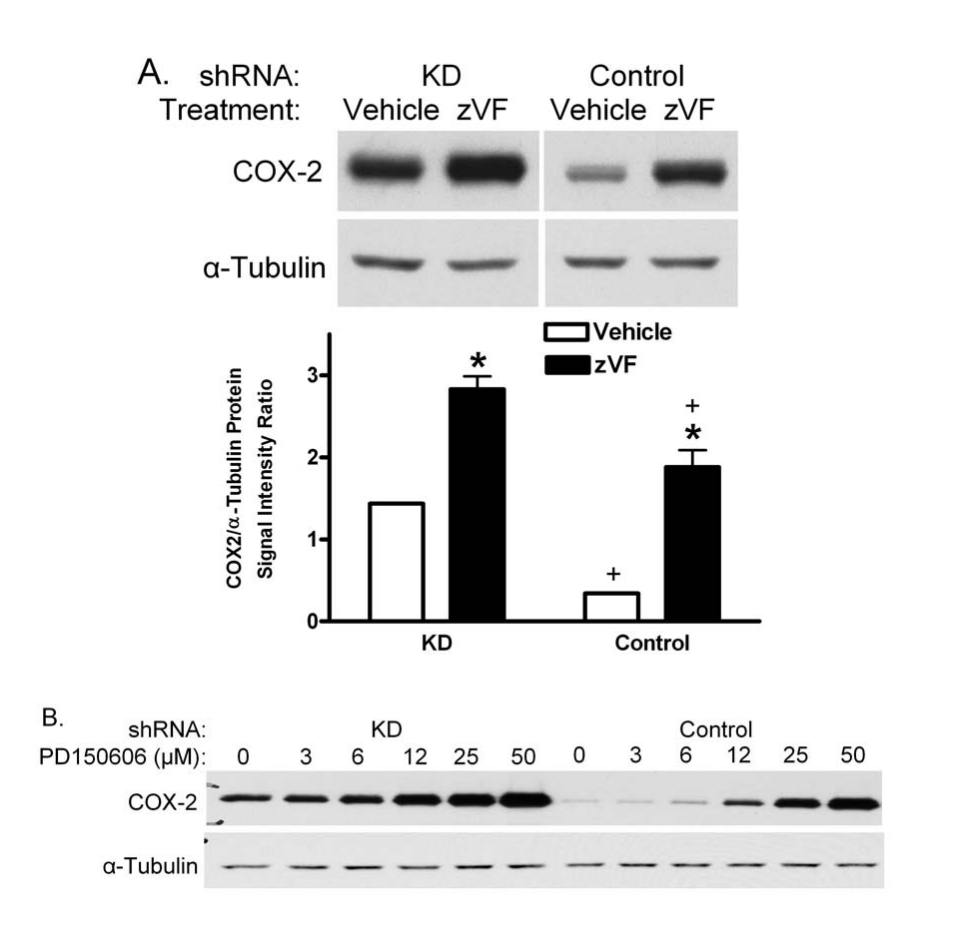
**Effect of calpain inhibitors on COX-2 protein**. PIN1 KD and Control shRNA MAEC were treated with vehicle (DMSO) or 25 μM zVF (A) or different concentrations of PD150606 (B), and then with LPS and IFN for 24 h. Representative western blots of COX-2 and α-tubulin are shown. Bars represent mean + SE ratio of COX-2 to α-tubulin from densitometric analysis of images from 3 independent cultures in each group. *: p < 0.05 for comparison between vehicle and zVF, and +, p < 0.05 for comparison between KD and Control shRNA.

**Figure 5:**
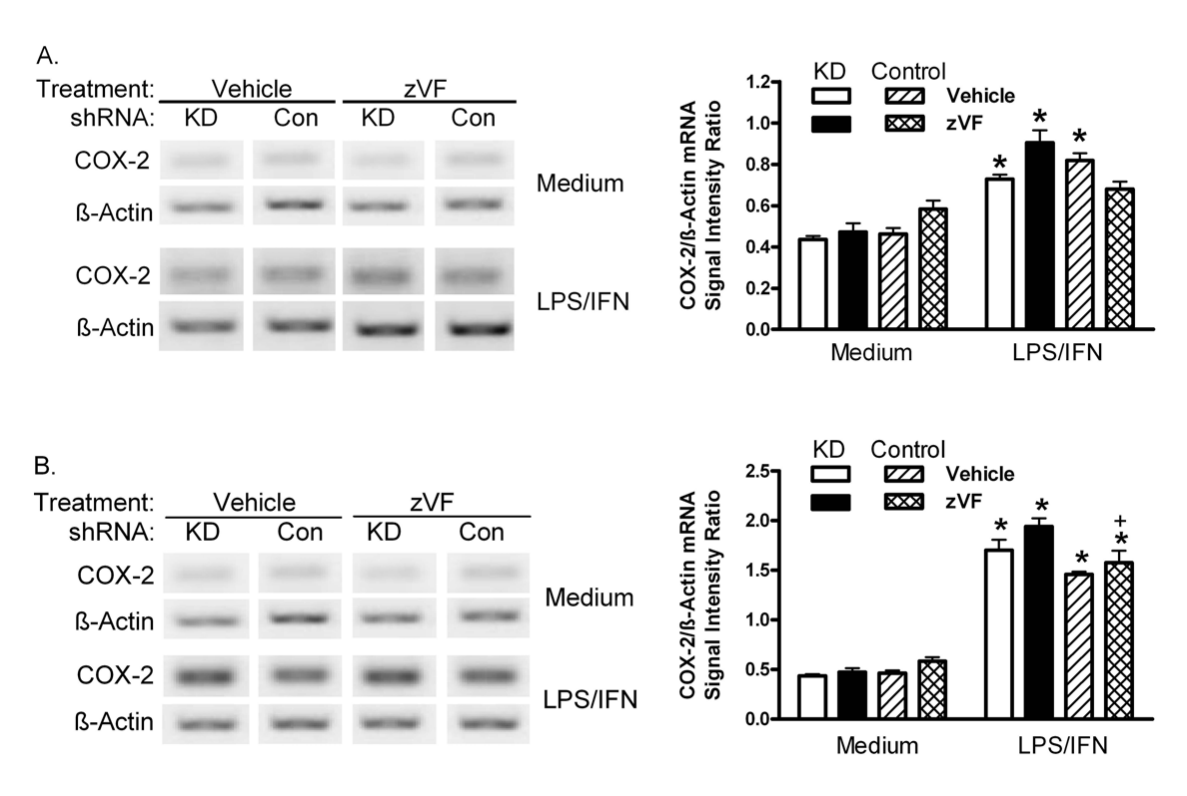
**Effect of zVF on COX-2 mRNA**. KD and Control (Con) shRNA MAEC were treated with vehicle (DMSO) or 25 μM zVF for 1 h, and then with medium or LPS/IFN for 1 h (A) or 24 h (B). mRNA for COX-2 and β-actin were assessed as in figure 3. Representative agarose electrophoresis of PCR products are shown. Replicate COX-2 or β-actin products in a single ethidium bromide-stained gel were imaged for analysis. Bars represent the mean + SE of ratio of COX-2/β-actin signal intensity + SE of 3 independent cultures. *: p < 0.05 for comparison between LPS/IFN- and medium-treated cells. +: p < 0.05 for comparison between KD and Control cells treated with LPS/IFN and zVF.

PIN1 KD and Control shRNA MAEC were pretreated with vehicle or the calpain inhibitors, zVF or PD150606, for 1 h, and then treated with LPS and IFN for 24 h. Again, COX-2 increased more in KD than Control cells (figure 4). In Control cells, COX-2 was induced 5.5-fold more in the presence of zVF than in its absence. zVF also increased the induction of COX-2 from its elevated level in KD MAEC by a factor of two (figure 4A). PD 150606, which is more selective than zVF for calpain relative to cathepsin activities [29,30], also increased the induction of COX-2 in KD and Control cells. zVF did not increase the induction of COX-2 mRNA in cells treated with LPS/IFN for 1 or 24 h (figure 5).

**Figure 6:**
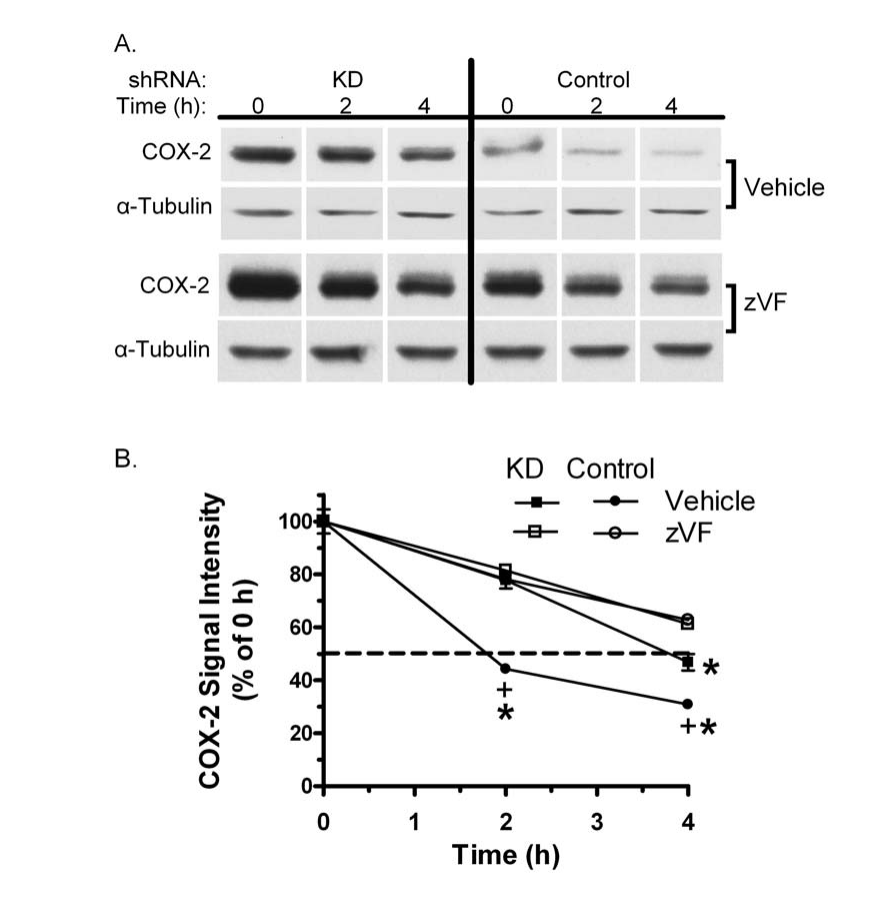
**Effect of PIN1 knockdown and calpain inhibition on COX-2 stability**. KD and Control shRNA MAEC were treated with vehicle (DMSO) or 25 μM zVF for 1 h, then with LPS and IFN for 24 h. Cycloheximide (90 μg/ml) was added, and cell extracts were collected at the indicated times and western blotted for COX-2 and α-tubulin. A, Representative images of COX-2 and α-tubulin. Blots were processed in the same reagents for each protein, and exposed on one film for all samples. B, The average COX-2/α-tubulin signal intensity ratio ± SE of 4 independent cultures for each point, as a percent of the value at 0 h after cycloheximide treatment, is shown. The dashed line marks the 50% value. *, p < 0.05 for comparison between vehicle- and zVF-treated KD cells or Control cells at the indicated time. +, p < 0.05 for comparison between similarly treated KD and Control cells at the indicated time.

The effect of PIN1 depletion and zVF on degradation of COX-2 was assessed. Cells were induced with LPS/IFN and then treated with 90 μg cycloheximide/ml to block translation. The level of COX-2 protein fell to 44% of its initial value 2 h after addition of cycloheximide to Control shRNA cells (figure 6). However, a similar decrease to 47% was delayed until 4 h in KD MAEC. COX-2 protein fell only to 78% of initial 2 h after cycloheximide in zVF-treated Control cells. zVF also inhibited the loss of COX-2 in KD cells at 4 h.

**Figure 7:**
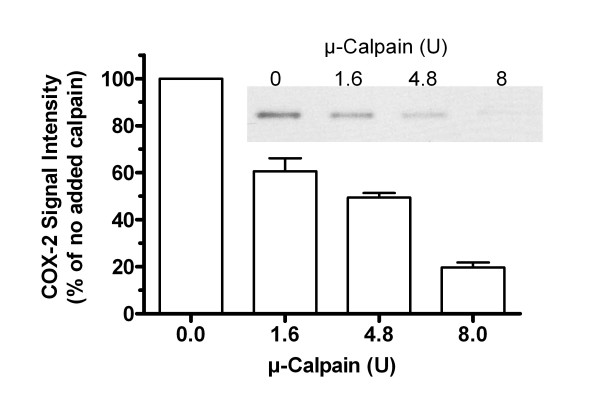
**COX-2 degradation by μ-calpain *in vitro***. Control cells were treated with LPS/IFN for 24 h to induce COX-2. Extracts were then mixed with the indicated units of porcine μ-calpain, in calpain reaction buffer, incubated 30 min, and then denatured for western blotting. A representative blot of COX-2 is shown. Bars represent the average COX-2 signal intensity ± SE of 3 independent cultures for each point, as a percent of the value without added calpain.

To confirm that COX-2 is a potential substrate for calpain, its digestion *in vitro *was examined. Addition of porcine μ-calpain to extracts of LPS/IFN-treated Control cells caused a concentration-dependent loss of COX-2 signal (figure 7).

Since PIN1 is known to bind its substrate proteins, interaction with COX-2 was investigated. Immunoprecipitation of PIN1 from extracts of vehicle- or LPS/IFN-treated Control cells did not produce any COX-2 detectable on western blots. COX-2 was not pulled down with glutathione-S-transferase-PIN1 fusion protein or glutathione-S-transferase (not shown).

**Figure 8:**
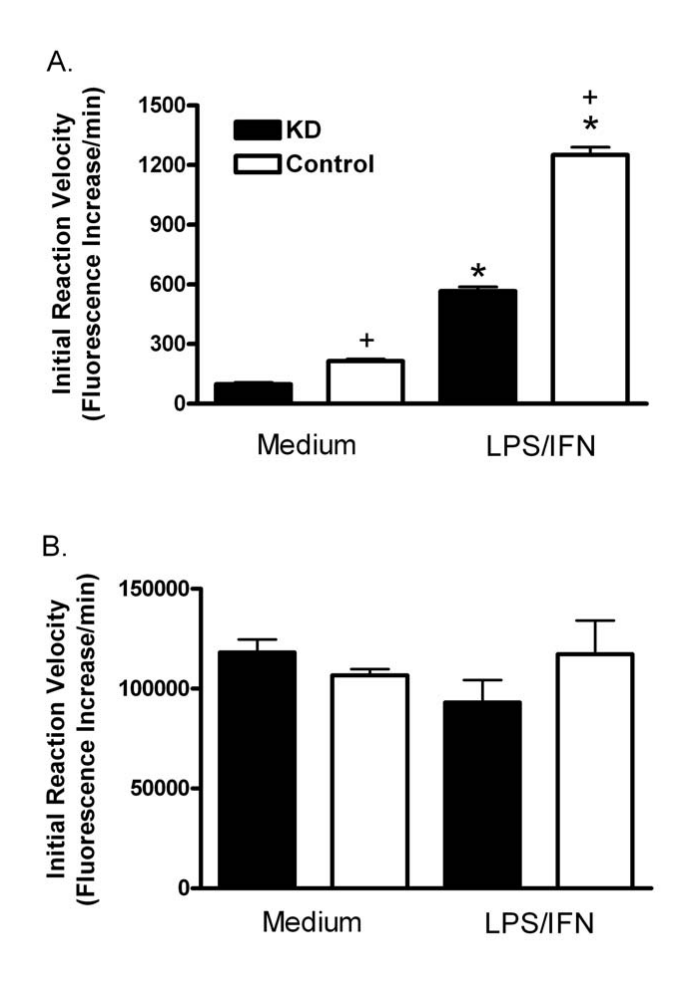
**Effect of PIN1 knockdown on calpain and cathepsin activity**. Activities were determined from the initial rate of cleavage of fluorogenic substrates for calpain (A), or cathepsin (B). These were assessed in extracts of KD and Control cells treated with medium or LPS/IFN for 24 h. Bars represent mean + SE fluorescence increase/min for 4 independent cultures in each group. *: p < 0.05 for comparison with medium-treated cells. +: p < 0.05 for comparison between KD and Control MAEC treated in the same way.

Given the effect of calpain inhibitors on COX-2, calpain and cathepsin activities were measured. LPS/IFN increased calpain activity 6.0-fold in KD cells, and 5.9-fold in Control MAEC. Calpain activity in vehicle-treated KD cells was approximately 45% of the activity in the Control cells with or without treatment with LPS/IFN (figure 8A). Cathepsin activity was measured since zVF might also inhibit it. There were no differences in cathepsin activity in extracts of KD and Control MAEC, with our without treatment with LPS/IFN (figure 8B).

## Discussion

PIN1 regulates the levels and activity of factors that can affect COX-2 synthesis in various cell types [2,4,31-36]. Here, suppression of PIN1 in endothelial cells increased the induction of COX-2, and COX-2-dependent production of prostaglandin E2 by LPS/IFN (figures 1 and 2). Despite a nearly 5-fold greater induction of COX-2 protein in KD compared with Control MAEC, there was no difference in the induction of COX-2 mRNA (figure 3). This suggests that PIN1 regulates COX-2 by a post-transcriptional mechanism. Consistent with a post-transcriptional effect, PIN1 depletion reduced the turnover of COX-2 (figure 6). Since COX-2 has a relatively short half-life, inhibition of turnover could lead to large, cumulative, post-transcriptional increases after induction with LPS/IFN [8].

One prior study revealed that cleavage of COX-2 was reduced by the inhibitor, E-64d, in human synovial fibroblasts [18]. Here, the calpain inhibitor, zVF, increased the induction of COX-2 (figure 4) and reduced its degradation (figure 6), without increasing its mRNA (figure 5). As for E-64d, zVF can also inhibit cathepsin activity at concentrations similar to those that inhibit calpain [29,37,38]. Therefore, PD 150606, which is more selective for calpain compared with cathepsin [30], was tested. Like zVF, PD 150606 increased the induction of COX-2 in KD and Control MAEC (figure 4B), further suggesting that calpain is responsible for restraining the induction of COX-2 in MAEC.

In support of this idea, it was shown here for the first time that PIN1 depletion reduced calpain activity in endothelial cells. In contrast, cathepsin activity was not affected by PIN1 depletion (figure 8). This result, combined with the effects of zVF and PD150606 on COX-2 induction and turnover, suggests again that calpain limits the expression of COX-2 in MAEC. Indeed, COX-2 was degraded by μ-calpain *in vitro*, indicating that it is a potential substrate in cells (figure 7). The reduced calpain activity in KD extracts (figure 8) could be due to an increase in expression or function of calpastatin or other unrecognized endogenous calpain inhibitors in KD cells, or to a reduction in expression or function of calpains [39]. Nevertheless, the results suggest that PIN1 depletion reduces calpain activity, consequently reducing the turnover of COX-2 in MAEC.

zVF further reduced the loss of COX-2 in cycloheximide-treated KD cells (figure 6). This may be due to the partial 55% reduction of calpain activity in KD MAEC (figure 8). The partially reduced calpain activity could account for the intermediate loss of COX-2 in the cycloheximide-treated KD cells, allowing zVF to further suppress turnover. It may also explain the ability of calpain inhibitors to increase induction of COX-2 in both KD and Control MAEC (figure 4). The partial reduction of calpain activity may be due to the incomplete (85%) suppression of PIN1 by the shRNA [6]. PIN1 may also function as a modulator of calpain activity and not as an absolute requirement.

Previously, we observed that PIN1 depletion and zVF each increased the induction of iNOS, and reduced its degradation. PIN1 physically interacted with iNOS. The WW and catalytic domains of PIN1 appeared to contribute to the association [6]. This suggested that PIN1 depletion might alter the susceptibility of its targets to digestion by calpain. For example, PIN1 could associate with these substrates and catalyze proline isomerization, affecting protease sensitivity. In contrast to iNOS, however, interaction between COX-2 and PIN1 was not detected here. A role for direct interaction between COX-2 and PIN1 cannot be completely excluded, however, since association of the proline isomerase with its putative substrate may be weak or transient. PIN1 could also affect association of COX-2, or iNOS, with other proteins that may indirectly regulate proteolysis. Thus, effects of PIN1 on calpain activity and/or COX-2, or associated factors, could affect the sensitivity of COX-2 to digestion with calpain.

Overall, the results indicate that PIN1 regulates the induction of COX-2, and iNOS, by a previously unknown effect on calpain-mediated turnover in MAEC. The mechanisms by which PIN1 regulates calpain activity are under investigation. In particular, PIN1 could affect the expression or activity of calpain subunits, and the endogenous inhibitor of heterodimeric calpains, calpastatin [39].

The effects of COX-2 in acute and chronic inflammatory responses in the vasculature are complicated by multiple primary and secondary stimuli that may be present, and by cellular factors, such as supply of arachidonic acid, complement of various prostaglandin synthases, and expression of prostaglandin receptors [16]. Here, depletion of PIN1 and inhibition of calpain each caused over-induction of both COX-2 and iNOS. The consequences of co-induction of these two particular enzymes may be significant. Peroxynitrite from NO increases prostaglandin synthesis [40], and S-nitrosylation of COX-2 activates the enzyme and contributes to cell injury [41,42]. The impact of the co-induction of iNOS and COX-2 in endothelium requires further investigation.

The role of calpain activity may also be complex. The most well-studied calpains, heterodimeric μ- and m-calpain, can cleave numerous protein substrates, and enhance or down-regulate different signal transduction processes. Excessive calpain activity can also cause cell injury and death in several organs, which can be reduced with calpain inhibitors [39,43]. Thus, it remains to be determined whether PIN1 or specific calpains in endothelial cells can be exploited to manipulate inflammatory activation in a therapeutically useful manner. In any case, the results here indicate that COX-2 is degraded by calpain, and that PIN1 regulates its expression via effects on calpain activity in MAEC.

## Conclusion

Depletion of PIN1 increased induction of COX-2 by LPS/IFN by a post-transcriptional mechanism associated with reduced calpain activity. Consistent with the short lifespan of COX-2 in MAEC, suppression of PIN1 and calpain inhibitors increased its induction. This previously unknown connection suggests that PIN1 may normally function to maintain calpain activity and, consequently, restrain the induction COX-2, iNOS, and perhaps other substrates in MAEC. PIN1 is likely to regulate a range of calpain-dependent endothelial activities.

## List of abbreviations

COX: cyclooxygenase; LPS: *E. coli *endotoxin; iNOS: inducible nitric oxide synthase; IFN: interferon-γ; KD: knockdown; MAEC: murine aortic endothelial cells; PIN1: Protein Never in Mitosis Gene A Interacting-1; shRNA: short hairpin RNA; zVF: carbobenzoxy-valinyl-phenylalaninal

## Competing interests

The authors declare that they have no competing interests.

## Authors' contributions

TL and DGH designed the study and collected results. RAS and RIL aided in cell culture, puromycin selection, and western blotting. VS, YH and LK were responsible for production of lentiviruses. TL and DGH were main authors and TL, RAS, VS, YH, LK and DGH edited the manuscript. All authors read and approved the final manuscript.
